# The impact of body mass index on treatment outcomes for patients with low-intermediate risk prostate cancer

**DOI:** 10.1186/s12885-016-2572-y

**Published:** 2016-07-29

**Authors:** Kosj Yamoah, Charnita M. Zeigler-Johnson, Abra Jeffers, Bruce Malkowicz, Elaine Spangler, Jong Y. Park, Alice Whittemore, Timothy R. Rebbeck

**Affiliations:** 1 Department of Radiation Oncology and Cancer Epidemiology, Moffitt Cancer Center & Research Institute, 12902 USF Magnolia Drive, Tampa, FL 33612 USA; 2 Jefferson Medical College and Kimmel Cancer Center of Thomas Jefferson University, Philadelphia, PA USA; 3 Stanford University School of Medicine, Stanford, CA USA; 4 The Abramson Cancer Center, University of Pennsylvania Perelman School of Medicine, Philadelphia, PA USA; 5 Dana Farber Cancer Institute and Harvard TH Chan School of Public Health, Boston, MA USA

**Keywords:** Body mass index, Biochemical failure, Tumor pathology, Prostate cancer treatment

## Abstract

**Background:**

Little is known about the relationship between preoperative body mass index and need for adjuvant radiation therapy (RT) following radical prostatectomy. The goal of this study was to evaluate the utility of body mass index in predicting adverse clinical outcomes which require adjuvant RT among men with organ-confined prostate cancer (PCa).

**Methods:**

We used a prospective cohort of 1,170 low-intermediate PCa risk men who underwent radical prostatectomy and evaluated the effect of body mass index on adverse pathologic features and freedom from biochemical failure (FFbF). Clinical and pathologic variables were compared across the body mass index groups using an analysis of variance model for continuous variables or χ^2^ for categorical variables. Factors related to adverse pathologic features were examined using logistic regression models. Time to biochemical recurrence was compared across the groups using a log-rank survivorship analysis. Multivariable analysis predicting biochemical recurrence was conducted with a Cox proportional hazards model.

**Results:**

Patients with elevated body mass index (defined as body mass index ≥25 kg/m^2^) had greater extraprostatic extension (*p* = 0.004), and positive surgical margins (*p* = 0.01). Elevated body mass index did not correlate with preoperative risk groupings (*p* = 0.94). However, when compared with non-obese patients (body mass index <30 kg/m^2^), obese patients (body mass index ≥30 kg/m^2^) were much more likely to have higher rate of adverse pathologic features (*p* = 0.006). In patients with low- and intermediate- risk disease, obesity was strongly associated with rate of pathologic upgrading of tumors (*p* = 0.01 and *p* = 0.02), respectively. After controlling for known preoperative risk factors, body mass index was independently associated with ≥2 adverse pathologic features (*p* = 0.002), an indicator for adjuvant RT as well as FFbF (*p* = 0.001).

**Conclusions:**

Body mass index of ≥30 kg/m^2^ is independently associated with adverse pathologic features, which is an indicator for additional RT, particularly in patients with low-intermediate risk disease. Future studies may determine if this select group of patients may be best treated with definitive RT to reduce toxicity from additional RT following radical prostatectomy. We propose including body mass index in clinical decision-making for appropriate treatment recommendation for patients with low-intermediate risk PCa.

## Background

Obesity, one of the most pressing issues facing the U.S. healthcare system, is a potentially modifiable risk factor for disease progression and poor outcomes for numerous diseases, including prostate cancer (PCa) [1]. Specifically, associations between increased body mass index (BMI) and advanced prostate tumor stage and grade at diagnosis, younger age at diagnosis, and biochemical failure (FFbF, disease recurrence) after treatment have been observed. [2–4] While investigators study the underlying mechanisms that link obesity to poor PCa outcomes [5–8], understanding how BMI may influence treatment recommendations is a critical aspect of ongoing PCa care.

The current guidelines for patients with organ-confined PCa include definitive modalities such as radical prostatectomy (RP) or radiation therapy (RT) [9–12]. RP is a standard surgical management for clinically localized PCa in patients free of surgical contraindications. This procedure confers excellent 10-year long-term disease control of >90 % in patients who are confirmed pathologically to have localized (pT2) disease. Retrospective studies reported that the long-term outcomes of patients with localized and low-risk PCa were equally favorable with RP or external beam radiation therapy [13, 14]. For intermediate and high risk disease, however, monotherapy with either RP or RT did not achieve the excellent long-term outcomes seen in patients with low-risk disease [15, 16]. For pT3 cancer (defined as disease in the extraprostatic extension or seminal vesicle involvement), the risk of 5-year local failure and biochemical progression varies from 20 % to 70 % [17, 18]. Several randomized studies for patients with pT3 (with or without positive margin) or pT2 (with positive surgical margin) disease have been reported, demonstrating that adjuvant RT reduces the risk of local relapse and biochemical progression and disease-specific survival [19–22]. Despite earlier cancer detection with serumPSA screening, approximately 50 % of patients who undergo RP are found to have at least one adverse pathologic feature(APF), including advanced tumor grade/stage and positive margins/lymph nodes, extraprostatic extension and seminal vesicle invasion [23]. These patients may require adjuvant RT.

Several studies have shown increased genitourinary and gastrointestinal toxicity from additional RT after RP [22, 24–26]. In the South West Oncology Group trial, adverse events were more likely to occur in the RP + RT arm compared with the RP arm (23.8 % *vs* 11.9 %), including urethral strictures (17.8 % *vs* 9.5 %), total urinary incontinence (6.5 % *vs* 2.8 %), and rectal complications (3.3 % *vs* 0 %), respectively [25]. A study on the health-related quality of life (HRQOL) of PCa patients compared short- and long-term effects of adjuvant treatment versus observation after RP [26]. The investigators reported that the addition of RT to RP resulted in more frequent urination, as well as early report of more bowel dysfunction. Another HRQOL in patients treated with multimodality for PCa reported a decline in HRQOL particularly with urinary function, urinary bother and sexual function [24]. Therefore, the ability to preoperatively identify the subset of patients who are at risk of requiring additional RT after RP will be of clinical utility. These patients may benefit from upfront definitive RT to improve quality of life and minimize additional toxicity from a combination of RP followed by RT. To date the most widely utilized predictors of clinical outcomes including PSA, Gleason score (GS) and clinical stage are sub-optimal in predicting adverse pathologic outcomes and adjuvant RT use following RP. Over the last decade, a large body of evidence has emerged associating obesity with incidence of PCa [27–29] as well as adverse outcomes following treatment. Recent studies found increased BMI to be associated with aggressive PCa and FFbF [30–34]. However, no studies have examined the relationship between preoperative BMI and the need for adjuvant RT following RP in patients with adverse pathologic outcomes. We sought to determine whether BMI provides a clinically useful prediction of adverse pathologic outcomes that will guide physicians in recommending RT for select patients with organ-confined PCa. Obesity, in particular, has been related to a number of factors and molecular pathways that may advance cancer progression [35]. We hypothesize that obesity status modifies the relationship between preclinical risk and PCa outcomes among low-intermediate risk patients. The study aims were to utilize a cohort of radical prostatectomy patients to 1) examine the relationship between obesity and adverse pathology, and 2) examine the relationship between obesity and FFBF.

## Methods

### Patient population

This study utilizes a cohort of 1970 men with PCa treated with RP and bilateral pelvic lymph node dissection at the Hospital of the University of Pennsylvania Health System (UPHS; Philadelphia, PA.) Patients were consented in person and recruited at UPHS to participate in a PCa study, the Study of Clinical Outcomes, Risk and Ethnicity (SCORE) between 1990 and 2012 as previously described [36, 37]. This study was approved by the Institutional Review Board at the University of Pennsylvania.

The SCORE study includes information on patient age, race, height, weight, clinical stage, clinical Gleason on diagnostic biopsy, preoperative PSA levels, surgical pathologic information (tumor grade, stage, surgical margins status, extraprostatic extension, or seminal vesicle involvement, lymph node status). Prospective follow -up was conducted with PSA levels obtained at each visit. For the purpose of this study, patients without height and weight data for BMI calculation were excluded from the analysis (*N* = 506). Patients without adequate preclinical data including initial PSA (*N* = 30), or biopsy Gleason (*N* = 264) at diagnosis were excluded from the analysis. Patients who received androgen deprivation therapy (ADT) or adjuvant RT and/or ADT were included. The remaining 1170 patients were analyzed in this study.

### Data collection

The standard protocol for men in the SCORE study was as follows: Patients were evaluated at time of diagnosis by a thorough history and physical examination (including digital rectal examination [DRE]) followed by routine laboratory studies, including serum PSA levels, and GS determined by needle biopsy and reviewed at the UPHS. The patients were examined 1 month postoperatively and then at 3 month intervals for 1 year, every 6 months for 5 years, and then annually. At each follow up visit a complete evaluation, including DRE and serial PSA values, were determined and recorded. Biochemical recurrence (PSA failure) was defined as a single PSA ≥0.2 ng/ml or when two consecutive PSA values of 0.2 ng/ml were obtained after an undetectable value. Time zero (the starting point for follow-up) was defined at the date of surgery for all patients. If PSA was never undetectable postoperatively, then PSA failure was assigned at time zero. Patients with no follow up data were included for the evaluation of differences in preoperative and pathologic characteristics, but not biochemical recurrence.

Data related to patient and clinical characteristics, tumor pathology, and PCa outcomes were collected via medical record abstraction. All patients were staged according to the 1992 American Joint Committee on Cancer staging system [38].

### Treatment

Surgical treatment consisted of a radical retropubic prostatectomy and bilateral pelvic lymph node sampling or robotic-assisted laparoscopic prostatectomy. Adverse pathologic features (APF), such as extraprostatic extension (EPE), seminal vesicle invasion (SVI), and surgical margin status (SM), were noted and recorded. At the discretion of the treating physician, patients with APF including EPE, SVI or positive surgical margins were treated with adjuvant RT and/or ADT. ADT consisted of a gonadotropin-releasing hormone agonist (leuprolide acetate or goserelin acetate) with or without antiandrogens (flutamide). The SCORE study is a prospectively maintained database with patients treated from the 1990s until 2012. For this reason the year of prostatectomy was recorded and introduced into our modeling to account for difference in pre-PSA era of diagnosis and improvements in surgical treatment techniques that may impact APFs.

### Risk classification

Preoperatively patients were stratified into low, intermediate and high risk groups according to the recent National Comprehensive Cancer Network (NCCN) guidelines [39]. Patients who had T1 to T2a tumors, and a Gleason score < 7, and a PSA level < 10 ng/mL were classified as low risk (*N* = 777); patients who had T2b to T2c tumors, and/or a Gleason score of 7, and/or a PSA level between 10 ng/mL and 20 ng/mL were classified as intermediate risk (*N* = 270); and patients who had > T3 tumors, or a Gleason score between 8 and 10, or a PSA level > 20 ng/mL were classified as high risk (*N* = 117) [38]. Following RP patients were further stratified by the number of APFs into low, intermediate and high RPrisk groups. Patients with no APFs were in the low RPrisk (*N* = 818); patients with only 1 APF were in the intermediate RPrisk (*N* = 177); and patients with >/=2 APFs were in the high RPrisk group (*N* = 175).

### Statistical analysis

#### BMI

For the purpose of this study BMI (weight in kilograms divided by height in meters squared) was categorized as follows; normal weight (<25 kg/m^2^), overweight (≥25 kg/m^2^ to <30 kg/m^2^), obese (≥30 kg/m^2^). BMI was examined as a continuous and a categorical variable in which case BMI was stratified into non-obese (<30 kg/m^2^) or obese (≥30 kg/m^2^).

#### Other patient/clinical variables

Age, PSA, year of surgery, and biopsy Gleason score were examined as continuous variables. Clinical T-stage (T1c, T2a, T2b, and T2c) and race (white, African-American/black, and other) were examined as categorical variables.

Clinical and pathologic variables were compared across the BMI groups using an analysis of variance model for continuous variables or χ^2^ for categorical variables. Factors associated with the presence of APF were examined using logistic regression models. The predictability of BMI was evaluated using more stringent criteria of ≥2 APF in order to rigorously select for the patients that are most likely to be offered additional RT. For Cox proportional hazards models predicting FFbF, patients who experienced biochemical recurrence or PSA failure, lost to follow up, deceased were censored. Treatment outcomes often correlate with biochemical control rates thus, a PSA rise to 0.2 ng/ml was used to define biochemical disease recurrence, and time to biochemical recurrence was used as a surrogate for biochemical disease-free survival. Time to biochemical recurrence was compared across the groups using a log-rank survivorship analysis. For both univariate and multivariate analyses, BMI, Race, clinical stage, and clinical Gleason score were evaluated as categorical variables as follows: BMI categorical used BMI <25 kg/m2 as reference category; Race used White as reference category; “Other” race was dropped from model due to small numbers. Clinical stage categorical (T1; T2a, T2b; >T2c), used T1c as reference category; clinical Gleason score categorical (6, 7, ≥8) used Gleason 6 as reference category. The analyses were conducted using STATA statistical software version 13.0 (STATA Corporation). A *p*-value <0.05 was considered statistically significant.

## Results

The patient clinical and pathologic characteristics are listed in Table 1. Preoperative factors such as age, PSA at diagnosis, biopsy Gleason, clinical T-stage and year of RP were similar between BMI categories except for race, where African American/Black race was associated with elevated BMI (*p* < 0.001). There was a statistically significant difference between postoperative pathologic features and BMI. Specifically, extraprostatic extension, *p* = 0.001; positive surgical margins, *p* = 0.01; and higher pathologic Gleason (*p* = 0.001).

**Table 1:** Pre- and post- treatment characteristics and pathologic outcomes of men undergoing radical prostatectomy at University of Pennsylvania, 1990–2012

	Body mass index (kg/m^2^)						*p-*value
	<25	%	25–30	%	>30	%	
iPSA (ng/ml)							0.95^b^
median	5.1		5.4		5.2		
mean	6.59		6.73		6.76		
IQR	3.9–6.8		4.2–7.2		4.1–7.2		
Age (years)							0.37^b^
median	60		60		59		
mean	59.1		59.2		58.6		
IQR	54–64		54–64		54–63		
Biopsy Gleason Score							0.46^a^
≤ 6	150	82	459	76	293	77	
7	25	14	102	17	61	16	
8 to 10	7	4	40	7	73	6	
Clinical T-stage							0.99^a^
T1c	121	80	392	81	251	80	
T2a	24	16	73	15	51	16	
T2b	3	2	9	2	6	2	
T2c	3	2	12	2	5	2	
Race							**<0.001** ^a^
white	149	83	487	82	270	71	
African-American/Black	31	17	106	18	112	29	
other	4	2	9	2	2	0.5	
Pathologic Gleason Score							**0.001** ^a^
≤ 6	106	58	321	53	165	43	
7	71	39	252	42	186	48	
8 to 10	7	4	29	5	33	9	
Nodal Status							0.47^a^
pN0	184	100	592	99	379	99	
pN1	0	0	3	1	3	1	
Extraprostatic spread	39	21	130	22	117	30	**0.004** ^a^
Seminal Vesicle invasion	9	5	29	5	26	7	0.39^a^
Positive surgical margin	19	10	108	18	78	21	**0.01** ^a^
Additional Radiotherapy	1	0.6	11	2	8	2	0.39^a^
Hormonal Therapy	7	4	30	5	16	4	0.51^a^
No. of patients	184		602		384		

NOTE. Boldfaced values represent statistically significant differences between groups

*Abbreviations*: *iPSA* initial prostate-specific antigen, *IQR* interquartile range

^a^ *P* value derived from Person’s chi-square test

^b^ *P* value derived from analysis of variance model

**Fig. 1:**
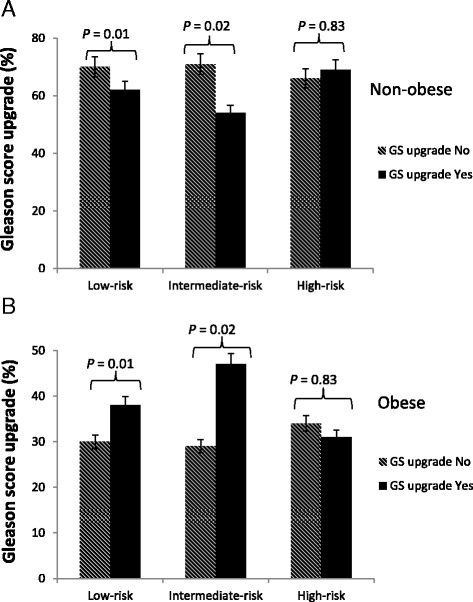
Rate of Gleason score upgrade in **a** Non-Obese and **b** Obese men undergoing radical prostatectomy at University of Pennsylvania, 1990-2012. Abbreviation: GS- Gleason score Gleason score upgrade represent upgrading from either score 6 or 7 to 8 – 10. Obese: ≤30kg/m2; Non-Obese <30kg/m2

As shown in Table 2, BMI was not associated with preoperative risk groupings (*p* = 0.94). However, obesity (BMI ≥30 kg/m^2^) directly correlated with increased risk of APFs (; *p* = 0.006). The effect of BMI and outcomes by pre-operative risk grouping was evaluated as per the National Comprehensive Cancer Network classification. Obesity (BMI ≥30 kg/m^2^) was strongly associated with a higher rate of pathologic Gleason score upgrading of tumors, particularly for low risk (Fig. 1a-b, 30 % vs. 38 %; *p =* 0.01) and intermediate risk patients (Fig. 1a-b, 29 % vs 47 %; *p =* 0.02).

**Table 2:** Correlation of body mass index with NCCN pre-operative risk grouping, and post-operative APFs in men undergoing radical prostatectomy at University of Pennsylvania, 1990–2012

	Body mass index (Kg/m^2^)				*p*-value
	≤30	%	>30	%	
Preoperative Risk group					0.94
Low	521	67	256	33	
Intermediate	184	68	86	32	
High	78	67	39	33	
Postoperative Risk group					**0.006**
APF = 0	571	70	247	30	
APF = 1	114	64	63	36	
APF ≥ 2	101	58	74	42	

*Abbreviations*: *NCCN* National Comprehensive Cancer Network, Obese: ≤30 kg/m^2^; Non-Obese <30 kg/m^2^

*APFs*: adverse pathologic features such as extraprostatic extension, seminal vesicle invasion, and/or positive surgical margin

Boldfaced values represent statistically significance

After controlling for known preoperative risk factors, BMI ≥30 kg/m^2^ was still predictive for the risk of having ≥2 APF (OR, 2.58; 95 % CI 1.30 to 5.09; *p* = 0.006) using BMI as a categorical variable with BMI <25 kg/m^2^ as the reference category (Table 3). Although African American/Black race is associated with elevated BMI, race was not associated with adverse pathologic outcomes in the logistic regression models.

**Table 3:** Univariate and multivariate regression models of factors predicting adverse pathologic outcomes in men undergoing radical prostatectomy at University of Pennsylvania, 1990–2012

Univariate analysis	OR	95 % CI	*p* value
Age	1	0.98 to 1.03	0.80
Race^a^			
*White*	1	Reference	
*African-American/Black*	1.21	0.83 to 1.77	0.32
Serum PSA	1.08	1.05 to 1.11	<**0.001**
Clinical stage^a^			
*T1c*	1	Reference	
*T2a*	1.05	0.64 to 1.72	0.85
*T2b*	3.00	1.11 to 8.17	**0.03**
*> T2c*	2.58	0.97 to 6.85	0.06
Year of Prostatectomy	0.97	0.94 to 1.00	0.05
Clinical Gleason score^a^			
*≤ 6*	1	Reference	
*7*	2.21	1.48 to 3.29	<**0.001**
*≥ 8*	5.06	3.03 to 8.43	<**0.001**
Body mass index, categorical^a^			
*< 25 kg/m2*	1	Reference	
*25 kg/m2 to <30 kg/m2*	1.48	0.86 to 2.53	**0.02**
*≥ 30 kg/m2*	2.20	1.27 to 3.81	**0.005**
Multivariate analysis			
Age	1	0.97 to 1.03	0.74
Race^a^			
*White*	1	Reference	
*African-American/Black*	0.81	0.50 to 1.32	0.40
Serum PSA	1.12	1.08 to 1.17	<**0.001**
Clinical stage^a^			
*T1c*	1	Reference	
*T2a*	1.24	0.72 to 2.14	0.43
*T2b*	2.28	0.70 to 7.38	0.17
*> T2c*	1.21	0.37 to 4.03	0.75
Year of Prostatectomy	0.99	0.95 to 1.04	0.69
Clinical Gleason score^a^			
*≤ 6*	1	Reference	
*7*	2.01	1.24 to 3.25	**0.005**
*≥ 8*	5.97	3.02 to 11.78	<**0.001**
Body mass index, categorical^a^			
*< 25 kg/m* ^*2*^	1	Reference	
*25 kg/m* ^*2*^ *to <30 kg/m* ^*2*^	1.58	0.81 to 3.07	0.18
*≥ 30 kg/m* ^*2*^	2.58	1.30 to 5.09	**0.006**

*Abbreviations*: *PSA* prostate-specific antigen, ≥2 adverse pathologic features as endpoint

^a^Denotes categorical variables. Body mass index, categorical uses BMI <25 kg/m^2^ as reference

Race use White as reference category; “Other” race was dropped from model due to small numbers

Clinical stage, categorical uses (T1; T2a, T2b; >T2c), with T1c as reference category

Clinical Gleason score, categorical (6, 7, ≥8); with 6 as reference category

*P* values derived from a logistic regression model

Boldfaced values represent statistically significance

**Fig. 2:**
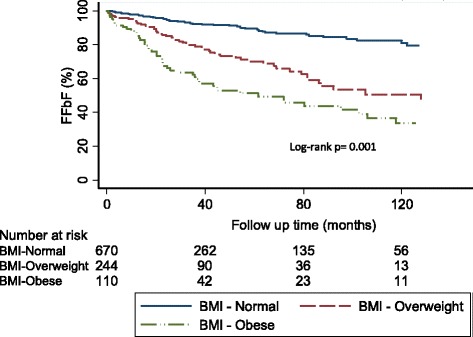
Kaplan-Meier curves for FFbF outcomes stratified by body mass index in men undergoing radical prostatectomy at University of Pennsylvania, 1990–2012. Abbreviations: FFbF- Freedom From biochemical Failure, NCCN- National Comprehensive Cancer Network, BMI- Body mass index, BMI- Normal: <25kg/m2; BMI - Overweight: ≥25 to <30 kg/m2; BMI- Obese: ≥30kg/m2

**Fig. 3:**
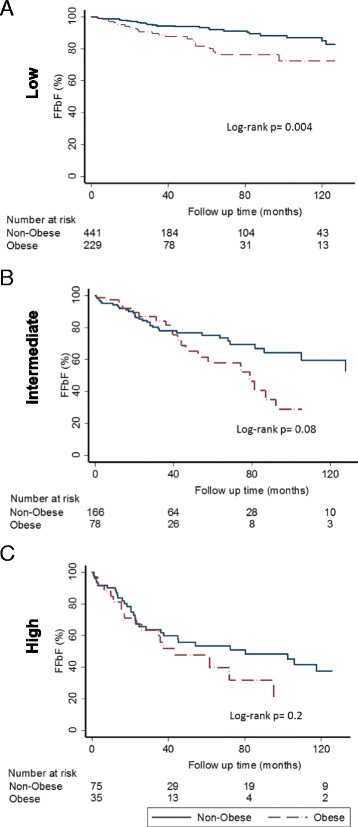
Kaplan-Meier curves for FFbF outcomes by BMI stratified by NCCN risk groups in men undergoing radical prostatectomy at University of Pennsylvania, 1990–2012. Abbreviations: FFbF- Freedom From biochemical Failure, NCCN- National Comprehensive Cancer Network, BMI- Body mass index, Non-Obese: <30kg/m2; Obese: ≥30kg/m2 . NCCN risk groupings: Panel A) Low risk; B) Intermediate risk; C) High risk

Using the Kaplan-Meier survival analysis method, the impact of BMI on freedom from (FFbF) was evaluated. The mean and median follow up time was 42 and 24 months, respectively (range 1–245 months). During this time period, 171 patients (15 %) experienced biochemical recurrence. Higher BMI was significantly associated with worse 7-year FFbF as follows; BMI-normal: 87 % vs. BMI-Overweight: 76 % vs. BMI-Obese: 61 %; log-rank *p* = 0.0004; Fig. 2. Upon stratifying by risk groupings BMI ≥30 kg/m^2^ had a significant impact on 7-year FFbF among patients with low risk (90 % vs. 76 %; log-rank *p* = 0.004), and a trend towards worse outcomes for intermediate risk disease (67 % vs. 40 %; log-rank *p* = 0.08), as shown in Fig. 3.

Using a multivariate Cox proportional hazard modeling, the significant predictors of risk for FFbF following RP were determined, Table 4. After adjusting for the other preclinical factors BMI ≥30 kg/m^2^ (HR, 2.56; 95 % CI 1.24 to 5.29; *p* = 0.01) remained a predictor of biochemical recurrence.

**Table 4:** Univariate and multivariate regression models of pre-operative factors predicting FFbF in men undergoing radical prostatectomy at University of Pennsylvania, 1990–2012

Univariate analysis	HR	95 % CI	*p* value
Age	1	0.98 to 1.03	0.80
Race^a^			
*White*	1	Reference	
*African-American/Black*	1.28	0.92 to 1.78	0.15
Serum PSA	1.03	1.02 to 1.04	<**0.001**
Clinical stage^a^			
*T1c*	1	Reference	
*T2a*	1.57	1.01 to 2.45	**0.04**
*T2b*	2.70	1.24 to 5.88	**0.01**
*> T2c*	0.72	0.18 to 2.92	0.64
Year of Prostatectomy	1.01	0.98 to 1.05	0.55
Clinical Gleason score^a^			
*≤ 6*	1	Reference	
*7*	2.34	1.61 to 3.39	<**0.001**
*≥ 8*	4.63	3.12 to 6.87	<**0.001**
Body mass index, categorical^a^			
*< 25 kg/m2*	1	Reference	
*25 kg/m2 to <30 kg/m2*	1.99	1.13 to 3.50	**0.02**
*≥ 30 kg/m2*	2.89	1.62 to 5.16	<**0.001**
Multivariate analysis			
Age	0.99	0.96 to 1.01	0.31
Race^a^			
*White*	1	Reference	
*African-American/Black*	0.67	0.43 to 1.04	0.07
Serum PSA	1.02	1.01 to 1.03	<**0.001**
Clinical stage^a^			
*T1c*	1	Reference	
*T2a*	1.75	1.11 to 2.75	**0.015**
*T2b*	0.91	0.36 to 2.30	0.84
*> T2c*	0.27	0.06 to 1.14	0.07
Year of Prostatectomy	1.02	0.99 to 1.06	0.20
Clinical Gleason score^a^			
*≤ 6*	1	Reference	
*7*	2.69	1.71 to 4.24	<**0.001**
*≥ 8*	7.27	4.26 to 12.42	<**0.001**
Body mass index, categorical^a^			
*< 25 kg/m* ^*2*^	1	Reference	
*25 kg/m* ^*2*^ *to <30 kg/m* ^*2*^	1.97	0.97 to 4.01	0.06
*≥ 30 kg/m* ^*2*^	2.56	1.24 to 5.29	**0.01**

*Abbreviations*: *PSA* prostate-specific antigen, *FFbF* freedom from biochemical failure

^a^Denotes categorical variables. Body mass index, categorical uses BMI <25 kg/m^2^ as reference

Race use White as reference category; “Other” race was dropped from model due to small numbers. Clinical stage, categorical uses (T1; T2a, T2b; >T2c), with T1c as reference category

Clinical Gleason score, categorical (6, 7, ≥8); with 6 as reference category

*P* values derived from a logistic regression model

Boldfaced values represent statistically significance

## Discussion

In this current study, further evidence was provided to suggest that BMI is a strong predictor of APF and biochemical recurrence following RP as monotherapy, particularly in patients with low- and intermediate-risk PCa. Specific groups of PCa patients with localized disease at presentation may be at increased risk for disease progression and related PCa-specific mortality from pre-treatment patient phenotype (e.g., obesity.) or post-treatment adverse pathologic features (e.g., positive surgical margins, seminal vesicle invasion, extracapsular extension). Multimodal treatment techniques have been employed to increase recurrence-free survival among localized high risk patients [40], and may be useful to treat a subset of patients with lower risk disease characteristics but elevated phenotypic risk factors. Although low- and intermediate risk patients often are treated with monotherapy, obese men are a patient population with unique disease features and medical needs that may require a more aggressive treatment approach including adjuvant RT.

### Obesity and PCa outcomes

Previous studies on the association between BMI the risk of developing PCa have provided mixed results. Obesity has been shown to increase the risk of poor PCa outcomes in several studies [2, 41–43]. Recent studies have analyzed the relation between BMI and PCa risk stratified by clinical stage and Gleason score at diagnosis. These studies consistently showed that elevated BMI positively correlated with increased risk of higher Gleason grade or higher stage disease and negatively correlated with low Gleason grade and stage of disease [27, 28, 44]. Previous results suggest that obesity is associated with higher grade tumors, increased risk of positive surgical margins, higher FFbF rates, and risk for PCa-specific mortality [1, 2, 33, 45–47]. In multivariate analyses, obesity also has been associated with significant tumor upgrading and upstaging among pre-operative low-risk patients, which increases risk for FFbF among this patient population [48–52].

However, not all studies support a relationship between poor PCa outcomes and obesity [53–55]. Often, studies differ by the number of obese men, sample population demographics and study methodology, making it difficult to compare across studies. Further complicating the relationship between obesity and PCa are diagnostic and treatment obstacles associated with obesity that make it more likely that cancer will progress and that treatments will fail on the obese patient due to technical difficulty rather than biological processes [41, 56–58]. Obese men are less likely than non-obese men to have abnormal PSA results and undergo biopsy, potentially effecting timely diagnosis. At the time of biopsy, larger prostate glands may make it more difficult to detect and accurately stage cancer [41, 59, 60]. It is also not clear if the relationship between obesity and treatment failure is due to aggressive disease biology or to technical limitations. Potency and continence rates after treatment are similar among weight groups, so technically inferior operations do not account fully for differences in treatment failure [1]. Pelvic surgery in general is more technically challenging in obese patients. Obesity has been associated with 30 % higher odds of capsular incision, a surrogate for poor technical operation. However, some patients receiving poor technique do well after surgery and others that experienced apparently fine surgical technique still experience FFbF [61].

### Treatment guidelines for low and intermediate risk patients

Treatment outcomes for patients with low- and intermediate risk disease have been inconsistent in part due to tumor heterogeneity and inaccuracies in staging [62, 63]. For this reason, low risk patients have recently been reclassified into very low-risk group (active surveillance eligible) and low-risk, and there are ongoing discussions to re-classify intermediate risk patients into low- and high- intermediate risk groups [64]. Therefore, the ability to preoperatively identify low or intermediate risk patients with elevated BMI who are at highest risk of FFbF after RP as monotherapy will be very useful in guiding upfront treatment recommendations. Perhaps, these patients may be best treated with combination therapy (surgery and RT), or other approaches such as definitive RT with/without hormonal therapy to improve disease control [40].

The current treatment guidelines recommend that patients with ≥1 APF be offered RT adjuvantly, or as part of a salvage regimen upon a detectable rise in PSA above 0.2 ng/ml following RP. Adjuvant RT has been shown in randomized trials to improve PSA-relapse free survival [19, 22, 65], distant metastasis-free survival and overall survival [66], compared to observation. Despite these results, referral patterns for additional RT for these patients remain very low. In fact less than 20 % of qualifying patients in the United States actually receive adjuvant RT [67–70] suggesting that many clinicians are reluctant to deliver RT after RP. In our study, only 2 % of entire cohort had documented treatment with additional RT. However, this result may be an underestimation and should be interpreted with caution, since a good number of patients could undergo RP at UPHS and then RT locally. RT information for these patients may not be accurately captured in our database. The primary reasons for withholding post-RP RT include increased treatment-related toxicity and potentially over treating patients with RT who may not recurred after RP [71]. It is estimated that approximately 50 % of patients with APF will not experience FFbF. Therefore, patients are offered “active surveillance” post-RP, and RT is only recommended at the earliest signs of PSA failure termed “early salvage”. However, whether early salvage RT is equivalent to adjuvant RT is a topic of current investigation [29]. Unlike RT post-RP, the use of ADT for patients in this setting is even much less standardized since physicians often recommend ADT for a number of reasons including attempting to reduce the prostate size prior to surgery or at the earliest signs of PSA failure after surgery.

Currently, the decision to recommend definitive radiation treatment for patients with low-intermediate risk prostate disease is often based on many factors including patient preference, and/or preexisting comorbid conditions that precludes surgery. However, in patients with no contraindications for surgery, decision for RT or RP is largely driven by age, genitourinary toxicity, and the desire to preserve sexual function [71]. The ability to identify patients with low-intermediate disease yet at increased risk for APF as well as FFbF will enable clinicians to better counsel patients with the appropriate treatment option that provides the best disease control with minimal side effects. The existing preclinical factors used to predict APF and biochemical outcomes are suboptimal. In this report elevated BMI was identified as a preclinical factor that is independently associated with adverse pathologic outcomes as well as biochemical recurrence, particularly in patients with low-intermediate disease or ≤ one adverse pathologic feature. Therefore, incorporating BMI into the current predictive models may shows promise in identifying the group of patients with low-intermediate risk disease who are likely to experience biochemical recurrence following RP as monotherapy. These patients could be best treated with definitive RT with or without hormones thus sparing them the added toxicity of requiring additional RT after RP. Further studies are required to develop and validate the predictive performance of BMI using an independent patient cohort.

### Study limitations

It is important to emphasize that results from this study cannot be extrapolated to imply that RP is suboptimal for obese men since not all patients with elevated BMI experience adverse pathologic outcomes or biochemical recurrence after surgery. Although elevated BMI was associated with increased positive surgical margins, BMI still was associated with adverse pathologic outcomes after adjusting for margin status. This suggests that poorer surgical outcomes did not account for worse pathologic outcomes in obese men. Therefore, patients with elevated BMI may harbor a biologically more aggressive PCa. Limitations to this study were that important measures of obesity, such as waist-to-hip ratio and percent lean body fat were unavailable. Information on the biologic factors that may contribute to the effect of elevated BMI on disease aggressiveness and treatment outcomes could not be evaluated since blood biosamples were not obtained at the time of surgery. Furthermore, the median follow-up for the study cohort was relatively short.

## Conclusions

Elevated BMI is independently associated with APF, particularly in patients with low-intermediate risk disease. BMI may be useful as a predictive tool to augment the performance of the known preclinical factors in predicting adverse pathologic outcome and the use of adjuvant RT post-RP. Therefore, BMI should be considered in clinical decision-making for appropriate treatment recommendation for patients with low-intermediate risk PCa.

## Abbreviations

ADT, androgen deprivation therapy; APF, adverse pathologic features; BMI, body mass index; DRE, digital rectal exam; EPE, extraprostatic extension; FFbF, biochemical failure; GS, gleason score; HRQOL, health-related quality of life; NCCN, National comprehensive cancer network; PCa, prostate cancer; PSA, prostate specific antigen; RP, radical prostatectomy; RT, radiation therapy; SCORE, study of clinical outcomes, risk and ethnicity; SM, surgical margin status; SVI, seminal vesicle invasion; UPHS, University of Pennsylvania health system
